# Prevalence of genotypic antimicrobial resistance in clinical Shiga toxin-producing *Escherichia coli* in Norway, 2018 to 2020

**DOI:** 10.1099/jmm.0.001454

**Published:** 2021-12-06

**Authors:** Silje N. Ramstad, Lin T. Brandal, Arne M. Taxt, Yngvild Wasteson, Jørgen V. Bjørnholt, Umaer Naseer

**Affiliations:** ^1^​ Department of Microbiology, Division of Laboratory Medicine, Oslo University Hospital, PB 4956 Nydalen, 0424 Oslo, Norway; ^2^​ Institute of Clinical Medicine, University of Oslo, Oslo, Norway; ^3^​ Department of Infectious Diseases and Prevention, Norwegian Institute of Public Health, Oslo, Norway; ^4^​ ECDC fellowship Programme, Public Health Microbiology path (EUPHEM), European Centre for Disease Prevention and Control (ECDC), Solna, Sweden; ^5^​ Department of Paraclinical Sciences, Norwegian University of Life Sciences, Oslo, Norway

**Keywords:** Shiga toxin-producing *Escherichia coli*, antimicrobial treatment, antimicrobials, whole-genome sequencing, genotypic resistance determinants

## Abstract

**Introduction:**

Shiga toxin-producing *Escherichia coli* (STEC) can cause severe to fatal disease in humans. Antimicrobial treatment is sometimes necessary, but contraindicated due to undesirable clinical outcome. However, recent studies have shown promising outcomes following antimicrobial treatment. Before the establishment of a possible antimicrobial treatment strategy for STEC infections, the prevalence of antimicrobial resistance in STEC needs to be determined.

**Gap statement:**

The resistance status of Norwegian clinical STEC is not known and should be assessed.

**Aim:**

We aim to characterize genotypic antimicrobial resistance determinants in clinical STEC in Norway, and determine the prevalence of genotypic resistance in order to inform possible antimicrobial treatment options for STEC infections.

**Methodology:**

We included all clinical STEC submitted to the Norwegian Reference Laboratory from March 2018 to April 2020. All samples were whole-genome sequenced and screened for genotypic antimicrobial resistance,virulence determinants and plasmid incompatibility groups. We performed phylogenetic clustering of STEC by core-genome multi-locus sequence typing, and statistical association analyses between isolate characteristics and genotypic resistance.

**Results:**

A total of 459 STEC were analysed. For 385 (83.9 %) STEC we did not identify any antimicrobial resistance determinants. Seventy-four STEC (16.1 %) harboured antimicrobial resistance determinants against one or more antimicrobial classes. The most frequent genotypic resistance was identified against aminoglycosides (10.5 %). Thirty-nine STEC (8.5 %) had a multi-drug resistance (MDR) genotype. Genotypic resistance was more prevalent in non-O157 than O157 STEC (*P*=0.02). A positive association was seen between genotypic resistance and the low-virulent STEC O117:H7 phylogenetic cluster (no. 14) (*P*<0.001). Genotypic resistance was not significantly associated to high-virulent STEC. STEC O146:H28 and isolates harbouring the plasmid replicon type IncQ1 were positively associated with MDR.

**Conclusion:**

The overall prevalence of genotypic resistance in clinical STEC in Norway is low (16.1 %). Genotypic resistance is more prevalent in non-O157 strains compared to O157 strains, and not significantly associated to high-virulent STEC. Resistance to antimicrobials suggested for treatment, especially azithromycin is low and may present an empiric treatment alternative for severe STEC infections.

## Introduction

Shiga toxin-producing *Escherichia coli* (STEC) is a zoonotic food- and waterborne pathogen of a serious public health concern because of its propensity to cause outbreaks, haemorrhagic colitis and potentially life-threatening complication haemolytic-uremic syndrome (HUS) [1–3]. The main reservoir of STEC is ruminants, such as cattle and sheep. Humans are infected with STEC through ingestion of faecally contaminated food or drinking water, through direct contact with carrier animals, or by person-to-person spread [4]. STEC is the third most common zoonotic infection within the EU [5].

Antimicrobials are presently contraindicated in the treatment of STEC infections, as there are indications that treatment could increase the risk of developing HUS, associated with increase in the Shiga toxin (Stx) production [6, 7]. However, recent studies have shown that it is possible to treat some STEC infections with certain classes of antimicrobials with a successful outcome [8–10]. Azithromycin, ciprofloxacin and fosfomycin (alone or in combination) are among the antimicrobials that have shown promising results in the treatment of severe STEC infections [8, 9, 11–13]. However, ciprofloxacin has shown to trigger Stx production *in vitro* [14–16]. Also, several *in vitro* studies investigating antimicrobials and their effects on Stx production have demonstrated that azithromycin, gentamicin and meropenem are potential candidates for treatment [14–16].

Parallel to investigations on the effects of different antimicrobial treatment options, it is important to consider the prevalence of antimicrobial resistance (AMR) in STEC. A review from 2018, reported prevalence of AMR in STEC between 8.6 and 62.5 % from different countries using a wide range of sampling and testing strategies [17]. Some studies have reported a higher prevalence of AMR in clinical non-O157 than O157 STEC [18–21].

Traditionally, determination of MICs of antimicrobials for a bacterial isolate is used to categorize clinical susceptibility or resistance. With the introduction of whole-genome sequencing (WGS), screening for the presence of resistance genes and chromosomal mutations known to confer resistance has become common for many pathogens [22, 23]. Studies investigating AMR in STEC by WGS has shown high concordance between phenotypic resistance and predicted AMR genotypes [18, 24, 25].

In Norway, all STEC infections are notifiable to the Norwegian Surveillance System for Communicable Diseases and microbiological isolates are sent to the National Reference Laboratory (NRL) for Enteropathogenic Bacteria at the Norwegian Institute of Public Health (NIPH). Over the last 5 years, on average 396 STEC cases have been notified annually (www.msis.no accessed 24 March 2021), of which 26 % were acquired abroad [26]. Approximately 300 (60 %) corresponding isolates have annually been submitted to the NRL. A detailed characterization of all STEC using WGS has been performed at NRL since March 2018 for outbreak detection and surveillance purposes [26].

In this study we characterize genotypic antimicrobial resistance determinants in clinical STEC isolated in Norway from 2018 to 2020, in order to determine the prevalence of genotypic resistance and inform possible antimicrobial treatment choice for STEC infections.

## Methods

### STEC isolates

All sporadic non-duplicate (one isolate per patient) clinical STEC isolates submitted to the NRL, as part of the national surveillance programme from March 2018 to April 2020 were included in this study. The STEC isolates were classified as high- or low-virulent based on the 2021 revised national guidelines for categorization of STEC [27]: STEC isolates identified with the Shiga-toxin-producing gene variants: *stx2a*, *stx2d* and/or *stx2c* were categorized as high-virulent, and all other STECs were categorised as low-virulent strains.

### Whole-genome sequencing

We extracted DNA using MagNAPure 96 (Roche Molecular Systems, Pleasanton, USA) and performed library preparation with KAPA HyperPlus (Kapa Biosystems, Wilmington, USA). Adapter dimers were removed by Agencourt AMPure XP (Beckmann Coulter Life Sciences, Indianapolis, USA), and Illumina technology (MiSeq or NextSeq, Illumina, San Diego, USA) was used to perform paired-end (250 bp ×2) sequencing aiming for coverage of >50×. FastQC (Babraham Bioinformatics, Cambridge, UK) was used for quality control of the raw reads. Assembly was done by SPAdes v3.13.2 with default settings. Quast was used for quality control of the assembly and Kraken was used for species identification. The sequences can be found under the project accession number PRJEB45863 at the European Nucleotide Archives (https://www.ebi.ac.uk/ena).

### Genomic analysis

We used the Center for Genomic Epidemiology’s (CGE) Bacterial Analysis Pipeline (BAP) with default threshold values (https://cge.cbs.dtu.dk/services/) for ResFinder 2.1, VirulenceFinder 1.2, MLST 1.6, PlasmidFinder 1.2 and SerotypeFinder 2.0, to identify resistance genes, serotypes, sequence types (STs), virulence genes and plasmid replicons [28] (last accessed 21 May 2020). Isolates that were not assigned a complete serotype through BAP were manually uploaded to the CGE SerotypeFinder 2.0. All isolates were run through the PointFinder database via an in-house pipeline for investigation of genotypic resistance due to known point mutations. We defined multidrug-resistant (MDR) STEC as the presence of resistance determinants to three or more antimicrobial classes [29]. We used SeqSphere+ 7.00.6 (Ridom GmbH, Münster, Germany) to perform core-genome multi-locus sequence typing (cgMLST) using the EnteroBase Warwick schema for *Escherichia */* Shigella *, followed by phylogenetic analysis by minimum-spanning tree (pairwise ignore missing values). Sequences with <90 % good targets in the cgMLST schema were excluded from the phylogenetic analysis. An outbreak cluster in the national surveillance programme is defined at ≤5 allelic differences (ADs) and a minimum of three isolates per cluster. For this study we defined phylogenetic clusters at ≤500 AD and a minimum 10 isolates per cluster.

### Statistics

We performed data analysis using Stata version 15.0 (StataCorp LP. USA). For each independent variable (*n*≥5); serotypes, virulence and plasmid replicon types, we estimated crude odds ratios (OR) and 95 % confidence interval (CI) for association to different classes of antimicrobials [quinolone, aminoglycoside, macrolide, sulphonamide, trimethoprim, tetracycline, ampicillin, extended spectrum cephalosporin (ESC)]. We calculated adjusted ORs (aOR) with 95 % CI in a multivariable analysis with binary outcome resistance markers/no resistance markers and MDR/non-MDR STEC. A two-tailed Fisher’s exact test was performed for prevalence comparison of genotypic resistance and O157/non-O157 and high-/low-virulent STEC. Similarly, the identified phylogenetic clusters were assessed for association to high-/low-virulent STEC and genotypic resistance. Also prevalence of MDR and O157/non-O157 STEC was assessed. A p-value of <0.05 was considered significant.

## Results

### Identification of outbreak clusters in clinical STEC

We analysed a total of 459 clinical STEC in this study, which were categorized into 12 outbreak clusters (*n*=61) and 398 sporadic strains. Five of the clusters contained high-virulent STEC, including four O157:H7 clusters (*n*=30, range 4–12 strains per cluster, all ST-11) and one O?:H2 cluster (*n*=5, ST-17). Seven of the clusters harboured low-virulent STEC, including three O103:H2 clusters (*n*=12, range 3–5 strains per cluster, all ST-17), two O63:H6 clusters (*n*=7, range 3–4 strains per cluster, both ST-583) and one cluster each of; O26:H11 (*n*=3, ST-21) and O142:H8 (*n*=4, ST-26), respectively.

### Antimicrobial resistance determinants in clinical STEC

In the majority of strains (83.9 %, *n*=385) no antimicrobial resistance determinants were identified. Only 74 strains harboured one or more resistance determinants (16.1 %) (Table 1). None of the strains identified with antimicrobial resistance determinants were part of any outbreak clusters. The most frequent genotypic resistance was identified against aminoglycosides (10.5 %, *n*=48) followed by sulphonamides (8.7 %, *n*=40), quinolones (8.1 %, *n*=37), tetracyclines (7.6 %, *n*=35) and ampicillin (6.1 %, *n*=28). Among the strains carrying antimicrobial resistance determinants, 39 (52.7 %) displayed a MDR genotype. We identified 34 different resistance genes and nine different point mutations known to confer phenotypic resistance against ten different antimicrobial classes (Table 1). The most common resistance genes identified were *strA* and *strB* (*n*=44), *aadA1* (*n*=12), *sul1* (*n*=14) *sul2* (*n*=38), *tetA* (*n*=31), *bla_TEM-1B_* (*n*=27) and *dfrA1* (*n*=13). Mutations in *gyrA* known to cause quinolone resistance were identified in 35 (7.6 %) strains. Only four strains harboured genes encoding ESC resistance, two *bla_CTX-M-15_* and two with point mutations in the *ampC* promoter region [30]. One strain had a point mutation in *rpoB,* which may cause resistance against rifampicin. No strains were identified with resistance determinants against carbapenems.

**Table 1.** Identified resistance determinants and proportions of resistance to the different antimicrobial classes in STEC (*n*=459) in Norway, 2018–2020

Antimicrobial classes	% of STEC genotypic resistant	Identified resistance determinants*
AMG	10.5 % (48)	*strA*, *strB* (*n*=44), *aadA1* (*n*=12), *aadA2* (*n*=5), *aadA5* (*n*=1), *aadA12* (*n*=1), *aadB* (*n*=1), *aac(3)-Iva* (*n*=2), *aph(3')-Ia* (*n*=11), *aph(4)]-Ia* (*n*=2), *aac(3)-Iid* (*n*=1)
SUL	8.7 % (40)	*sul1* (*n*=14), *sul2* (*n*=38)
QUI	8.1 % (37)	*QnrB19* (*n*=1), *QnrS1* (*n*=1), *QnrS2* (*n*=1), *gyrA* mutations p.S83L (*n*=75), p.D87N (*n*=1), p.D87Y (*n*=1), *parC* mutations p.S80I (*n*=1), p.S80R (*n*=1), p.A56T (*n*=1), *parE* mutation p.I355T (*n*=13)
TET	7,6 % (35)	*tet(A*) (*n*=31), *tet(B*) (*n*=5), *tet(M*) (*n*=1)
AMP	6.1 % (28)	*blaTEM-1B* (*n*=26), *blaTEM-1A* (*n*=1), *blaTEM-1C* (*n*=1)
TMP	4.4 % (20)	*dfrA1* (*n*=13), *dfrA5* (*n*=6), *dfrA8* (*n*=1), *dfrA12* (*n*=4)
MAC	2.4 % (11)	*mph(A*) (*n*=4), *mph(B*) (*n*=7), *erm(B*) (*n*=2)
PHC	2.4 % (11)	*floR* (*n*=9), *catB3* (*n*=1), *catA1* (*n*=2), *cmlA1* (*n*=1)
ESC	0.9 % (4)	*blaCTX-M-15* (*n*=2), *ampC* promoter mutation n.-42C>T (*n*=2)
RIF	0.2 % (1)	*rpoB* mutation p.Q513P
**Total**	**16.1 % (74**)	**43 different resistance determinants**

*No resistance detected against carbapenems, colistin, fosfomycins, fusidic acid, glycopeptides, nitroimidazole, or oxazolidinones. Aminoglycoside (AMG), sulphonamide (SUL), tetracycline (TET), ampicillin (AMP), trimethoprim (TMP), macrolide (MAC), phenicol (PHC), quinolone (QUI), extended-spectrum cephalosporine (ESC) and rifampicin (RIF).

### Serotypes, sequence types and resistance determinants in clinical STEC

The 459 strains were *in silico* typed into 92 different serotypes. The most common serotypes (*n*>20) included: O157:H7 (*n*=71, 15 %), O26:H11 (*n*=43, 9 %), O103:H2 (*n*=31, 6 %) and O146:H21 (*n*=22, 5 %). Forty-two strains were not assigned a complete serogroup. Similarly, the strains were typed into 81 different sequence types (STs). The most common STs (*n*>20) included: ST-11 (*n*=66, 14 %), ST-17 (*n*=37, 8 %), ST-21 (*n*=34, 7.5 %), ST-442 (*n*=23, 5 %) and ST-25 (*n*=22, 4.8 %). Eighteen strains were not assigned a known ST.

We identified antimicrobial resistance determinants in 28 different serotypes while six of the resistant strains were not assigned a complete serotype (Table 2). Among the strains of non-O157:H7 serotypes, 18.2 %(63 of 346) harboured resistance determinants against one or more antimicrobials, while 7 % (5 of 71) of the O157:H7 harboured resistance determinants (*P*=0.02). All STEC O117:H7 (*n*=10) were found to harbour resistance determinants to one or more antimicrobials. All STEC O80:H2 (ST-301, *n*=4) were genotypic resistant to aminoglycosides, sulphonamides, tetracyclines and ampicillin. Of serotype O26:H11 (*n*=43), eight strains harboured resistance determinants against one or more antimicrobials (ST-21 *n*=5, ST-29 *n*=3, 18.6 %), followed by O157:H7 (*n*=71) were five strains harboured resistance determinants (ST-11, 7 %) and O111:H8 (*n*=6) were four strains harboured resistance determinants (ST-16, 66.7 %).

**Table 2.** Numbers and proportions of identified resistance genotypes in most frequent serotypes and corresponding sequence types of STEC strains in Norway, 2018–2020

Serotype (total no.)	AMR* genotype												
	No.	%	AMG	SUL	TET	AMP	TMP	MAC	PHC	QUI	ESC	RIF	Sequence type
O146:H28 (11)	11	100								11			ST-738 (*n*=11)
O117:H7 (9)	9	100	4 3 1	5 3 1	4 2 1	3 1 1	2 3 1	1 1 1		2 2 1			ST-504 (*n*=5) ST-5292 (*n*=3) ST-6880 (*n*=1)
O26:H11 (43)	8	18.6	4 3	3 2	1 2	3 2			1				ST-21 (*n*=5) ST-29 (*n*=3)
O157:H7 (71)	5	7.0	5	5	3	4	5	3	5	2			ST-11 (*n*=5)
O111:H8(6)	4	66.7	3	3	4	3	3	3		3			ST-16 (*n*=4)
O80:H2 (4)	4	100	4	4	4	4	3		2	4			ST-301 (*n*=4)
O91:H14 (16)	3	18.8	2	2	3		1		2				ST-33 (*n*=3)
O128ab:H2 (14)	2	14.3	2	1	1	1	1	1		1			ST-25(*n*=2)
O80:H9 (4)	2	50									1 1		ST-23 (*n*=1) Unknown ST (*n*=1)
O55:H12 (3)	2	66.7	2	2	2	2				1			ST-101 (*n*=2)
O113:H4 (9)	1	11.1										1	ST-10 (*n*=1)
O24:H4 (1)	1	100	1	1	1						1		ST-117 (*n*=1)
O96:H19 (1)	1	100								1	1		ST-99 (*n*=1)
Other† (225)	15	–	10	6	4	4				5			Different STs
Unclassified‡ (42)	6	–	4	2	3		1	1	1	4			Different STs
Total (459)	74	16.1	48	40	35	28	20	11	11	37	4	1	

*Antimicrobial resistance (AMR)

†Other serotypes (genotypic resistance): O128ac:H10, O128ac:H8, O177:H25, O24:H4, O71:H2, O81:H21, O86:H2 and O96:H19 (*n*=1 for each), and other sequence types: ST-10, ST-448, ST-659, ST-117, ST-17, ST-737, ST-349 and ST-99 (*n*=1 for each).

‡Unclassified: lacking either the O-group, H-type or both.

Aminoglycoside (AMG), sulphonamide (SUL), tetracycline (TET), ampicillin (AMP), trimethoprim (TMP), macrolide (MAC), phenicol (PHC), quinolone (QUI) and extended-spectrum cephalosporin (ESC) and rifampicin (RIF).

### High-virulent strains and resistance determinants in clinical STEC

In total 35.5 % (*n*=163) of the STEC strains were classified as high-virulent. There was no significant difference in the presence of antimicrobial resistance determinants in high-virulent compared to low-virulent STEC (*P*=0.43). Of the high-virulent strains, 14.1 % (*n*=23) carried resistance determinants against one or more antimicrobials compared to 17.2 % (*n*=51) of the low-virulent strains (Table 3). All the strains carrying genes or point mutations known to cause ESC resistance were low-virulent.

**Table 3.** Number and proportions of identified genotypic resistance determinants to ten antimicrobial classes in high- and low-virulent STEC in Norway, 2018–2020

	Genotypic resistance					
	High-virulent STEC (*n*=163)		Low-virulent STEC (*n*=296)		All strains (*n*=459)	
Antimicrobial classes	No.	%	No.	%	Total	% all
All classes	23	14.1	51	17.2	74	16.1
AMG	20	12.3	28	9.5	48	10.5
SUL	17	10.4	23	7.8	40	8.7
QUI	11	6.7	26	8.8	37	8.1
TET	15	9.2	20	6.8	35	7.6
AMP	15	9.2	13	4.4	28	6.1
TMP	11	6.7	9	3.0	20	4.4
MAC	6	3.7	5	1.7	11	2.4
PHE	7	4.3	4	1.4	11	2.4
ESC	0	0	4	1.4	4	0.9
RIF	0	0	1	0.3	1	0.2
MDR	16	9.8	23	7.8	39	8.5

Aminoglycoside (AMG), sulphonamide (SUL), tetracycline (TET), ampicillin (AMP), trimethoprim (TMP), macrolide (MAC), phenicol (PHC), quinolone (QUI), extended-spectrum cephalosporin (ESC) and rifampicin (RIF).

### Multidrug resistance in clinical STEC

**Fig. 1.**
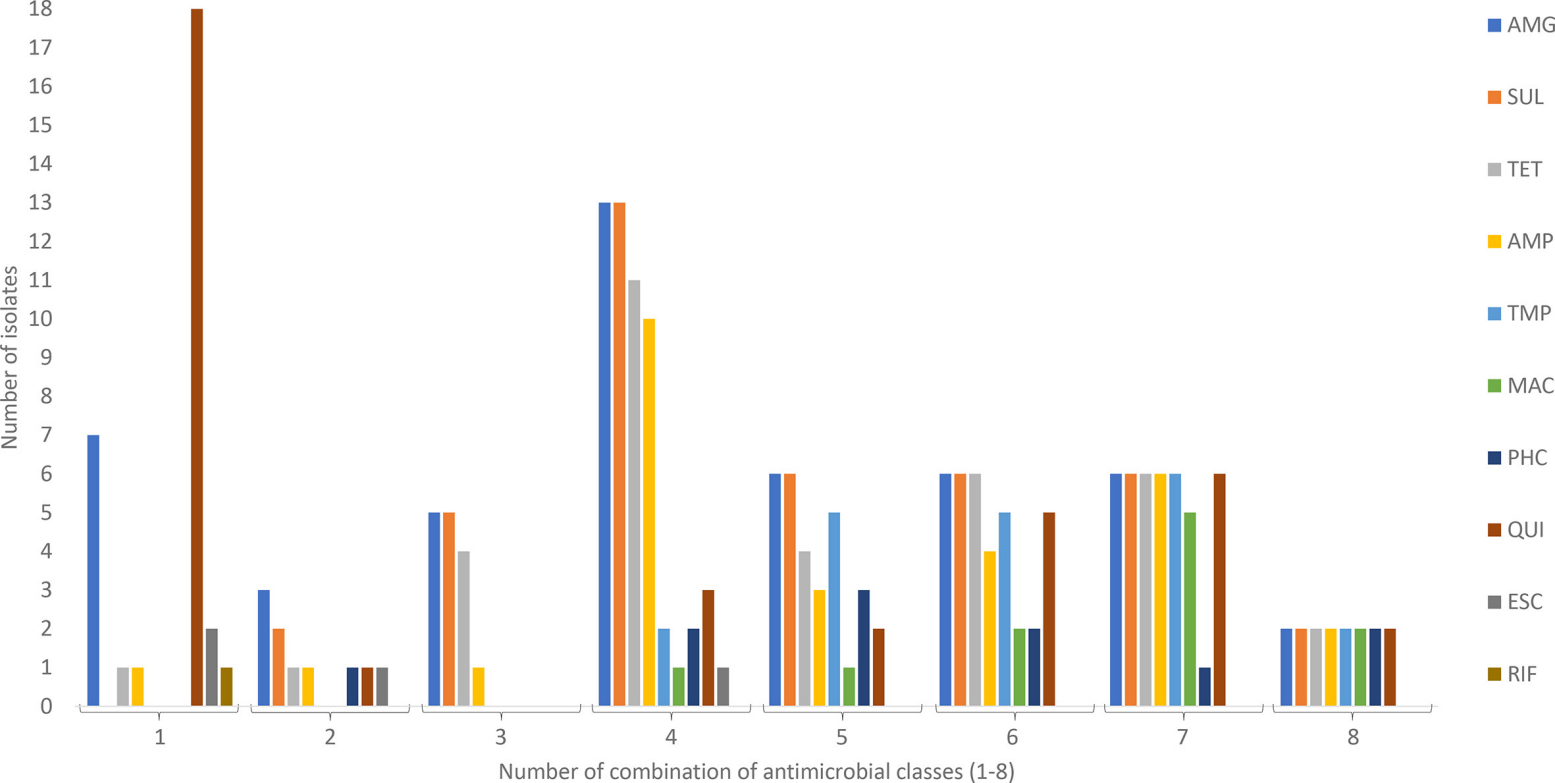
Number of STEC carrying molecular resistance determinants for 1 to 8 different classes of antimicrobials. The most frequent resistance determinants identified were against aminoglycoside, sulphonamide and quinolones. Thirty-nine strains were multidrug-resistant (MDR), conferring resistance against three or more antimicrobials. The most common combination among MDR STEC was antimicrobial resistance against aminoglycoside, sulphonamide and tetracycline, followed by resistance to quinolones, ampicillin and trimethoprim. Aminoglycoside (AMG), sulphonamide (SUL), tetracycline (TET), ampicillin (AMP), trimethoprim (TMP), macrolide (MAC), phenicol (PHC), quinolone (QUI) and extended-spectrum cephalosporine (ESC).

Approximately half of the strains carrying resistance determinants, 53 % (39 of 74), were MDR. Of the non-O157 strains with an assigned serotype, 9 % (31 of 346) were MDR, while 7 % (5 of 71) of the O157 strains were MDR (*P*=0.82). The most common combination of resistance determinants was against aminoglycosides, sulphonamides and tetracyclines (*n*=31) (Fig. 1). Five strains harboured resistance determinants against three different antimicrobial classes, 14 strains against four classes, six strains against five classes, six strains against six classes, and six strains against seven antimicrobial classes. Two high-virulent STEC O157:H7 encoded resistance against eight antimicrobial classes; aminoglycosides, tetracyclines, macrolides, sulphonamides, trimethoprim, phenicols, ampicillin and quinolones. There was no significant difference in the presence of MDR in high-virulent (9.8%) compared to low-virulent (7.8 %) STEC (*P*=0.49).

### Replicon-types identified in clinical STEC

We identified 41 different replicon-types (Table S1, available in the online version of this article). The most common replicon-types were IncFIB (*n*=345), Col (*n*=191) and Col156 (*n*=117). Thirty-four of these replicon types were identified in strains harbouring antimicrobial resistance determinants and 33 of these were identified in MDR STEC. In one O87:H16 STEC strain harbouring resistance determinant for quinolone resistance (*qnrB19),* no replicons were identified.

### Statistical associations between genotypic resistance and clinical STEC

In univariable analysis we observed a significant association between serotype O146:H28 (*n*=11) and genotypic resistance against quinolone (Table S2). Also, a positive association was observed between O111:H8 (*n*=6) and genotypic resistance to all main classes of antimicrobials, with the strongest association observed with tetracycline resistance OR 27.2 (95 % CI: 3.7–305). Among identified virulence genes, the strongest positive associations was seen for *capU* (*n*=15) and genotypic sulphonamide resistance OR 89.4 (95 % CI: 18.1–836), *sigA* (*n*=14) and genotypic aminoglycoside resistance OR 68.2 (95 % CI: 14.1–636), *iroN* (*n*=13) and genotypic beta-lactam resistance OR 15.1 (95 % CI: 3.8–56.2) and *espP* (*n*=134) and genotypic resistance against phenicol OR 6.8 (95 % CI: 1.6–40, Table S2). Among identified Inc-groups, the strongest positive associations were seen for IncQ1 (*n*=16) and genotypic resistance to the three antibiotic classes; aminoglycoside, beta-lactam and sulphonamide, where all strains were resistant. In addition, all but one strain harbouring IncQ1 had resistance determinants against tetracycline (OR 317.3, 95 % CI 42.96–13 377.68, Table S2). For other Inc-groups the strongest positive associations was seen for IncFIIpRSB10 (*n*=24) and genotypic macrolide resistance OR 28.7 (95 % CI: 6.5–127), ColBS512 (*n*=12) and genotypic macrolide resistance OR 31.4 (95 % CI: 5.5–152), IncB/O/K/Z (*n*=174) and genotypic sulphonamide resistance OR 7.8(95 % CI: 3.4–20), and IncFII (*n*=157) and genotypic beta-lactam resistance OR 3.64 (95 % CI: 1.6–8.7) (Table S2).

In multivariable analysis we observed that all strains serotyped O146:H28 or harbouring IncQ1 were genotypic resistant. In addition, a positive association was seen between *sigA* aOR 15.5 (95 % CI: 1.6–148) and *capU* aOR 9.4 (95 % CI: 1.5–60) and genotypic resistance. All strains harbouring IncQ1 were MDR and a positive association was seen between MDR genotype and IncFIIpRSB10 (95 % CI: 1.40–21.10) and IncBOKZ (95 % CI: 1.54–8.34). In addition, a positive association was seen between *capU* aOR 19.8 (95 % CI: 3.1–130) and *sigA* aOR 12.3 (95 % CI: 1.8–86) and a MDR genotype (Table S3).

### Phylogenetic clustering of clinical STEC

**Fig. 2.**
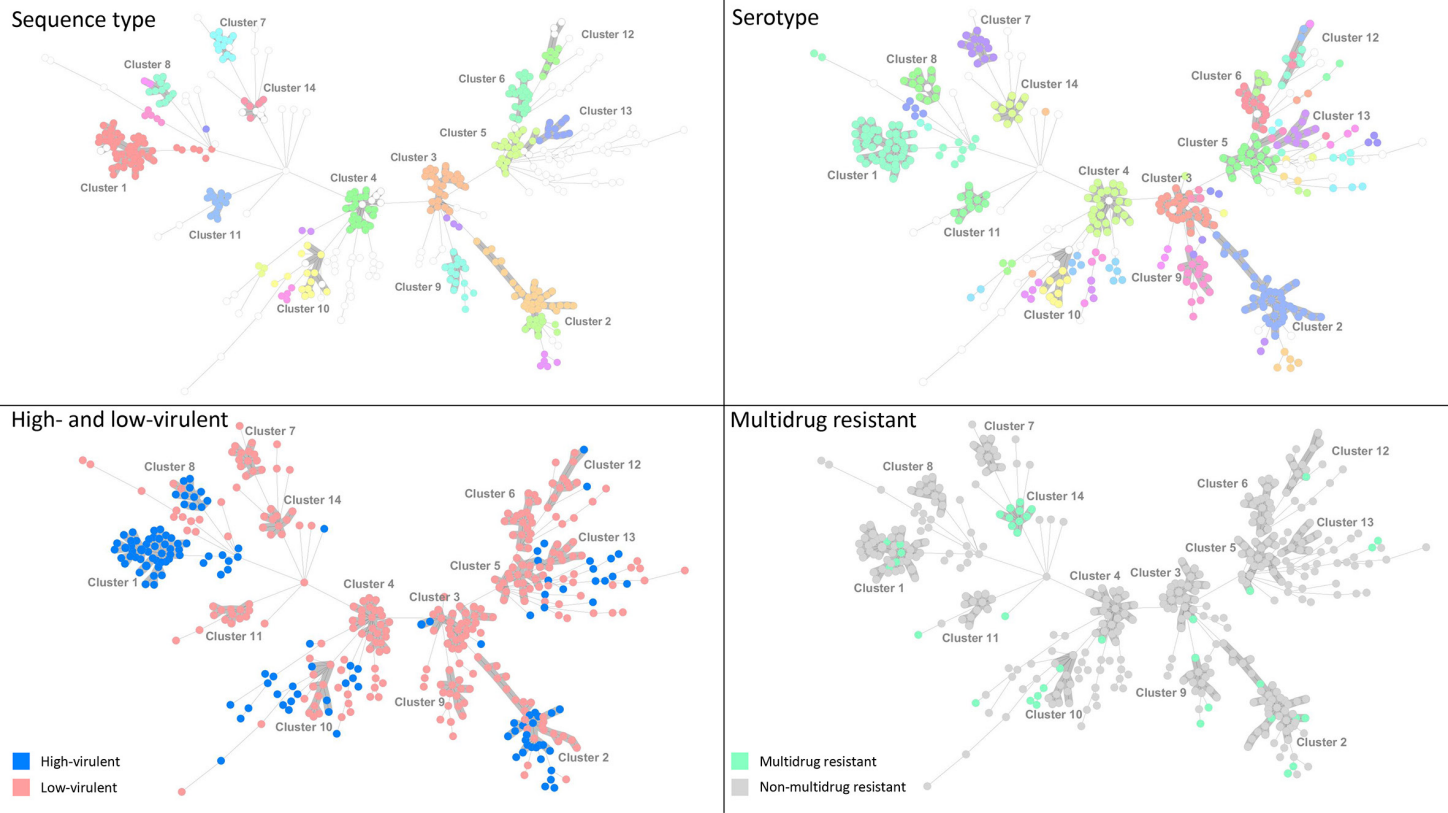
Minimum-spanning tree of STEC (*n*=456) stratified by ST, serotype, high- and low-virulent STEC, and MDR, Norway, 2018–2020. Strains are grouped into 14 phylogenetic clusters (≤500 allelic differences≥10 strains). See Table S4 for more details.

Sequences from three STEC were excluded from the phylogenetic analysis due to low quality. Using the set cluster definition (≥500 AD, ≥10 strains) we identified 14 phylogenetic clusters (*n*=318) ranging from 10 to 64 strains (Fig. 2). One-hundred and thirty-eight STEC were not assigned a cluster (singletons). The phylogenetic clustering of clinical STEC are summarized in Table S4.

Eleven clusters were significantly associated with low-virulent, and three clusters with high-virulent STEC (*P*<0.05). Only a single cluster, cluster 14, was positively associated with genotypic resistance (*P*<0.001). A small cluster consisting of four STEC O80:H2 (ST-301), not included in our cluster definition (<10 strains), was high-virulent and MDR. None of the STEC O80:H2 had the same resistance-gene-profile, even though many of the same genes were found [*strA*, *strB*, *aph(3')-Ia*, *sul2*, *tet(A*) and *bla_TEM-1B_*] and all strains had point mutations conferring quinolone resistance.

## Discussion

In this study we investigated the prevalence of genotypic resistance in clinical STEC in Norway (2018–2020) to inform possible antimicrobial treatment choice for STEC infections. We did not perform phenotypic antimicrobial susceptibly testing and AMR results are based on genotypic results only.

According to the Norwegian Surveillance System for Antimicrobial Drug Resistance (NORM), the prevalence of AMR in both human clinical samples and food-producing animals in Norway is among the lowest in Europe [31]. The overall majority (>90 %) of *E. coli* screened from non-human sources are susceptible to all tested antimicrobials. Also, antimicrobial usage in humans and animals in Norway is among the lowest in Europe [31]. However, there has been an increase of ESC resistance over the last 5 years, especially in *E. coli* isolated from cattle, but also in human clinical *E. coli* strains (blood cultures), where ESC resistance is now considered a significant clinical problem, with a reported prevalence of 7.1 % in 2019 [31].

We calculated the overall prevalence of genotypic resistance in clinical STEC to 16.1, 18.2 % among non-O157 and 7 % among the O157 STEC strains. This includes genotypic resistance to any of the screened antimicrobial classes. A similar report from the UK reported a prevalence of genotypic resistance as 27.3 % in non-O157 and 17.4 % in O157 strains [18, 25]. A higher prevalence of AMR in non-O157 STEC than in O157 STEC has also been reported from various phenotypic studies [19, 20, 32]. Also, we observed a higher percentage of MDR among the non-O157 strains compared to O157 STEC (8.8 and 7 %), although this was not significant.

We observed a positive association between genotypic resistance and the low-virulent STEC O117:H7 phylogenetic cluster (no. 14) (*P*<0.001). Previously, STEC O117:H7 has been associated with an outbreak of sexually transmitted enteric infection among men who have sex with men (MSM) in the UK [33]. The majority of the strains in cluster 14 were isolated from men (80%) and two of the strains clustered together with strains from the UK MSM outbreak (2013–2014). The outbreak strain was shown to harbour a large resistance plasmid containing *sul2*, *strA* and *strB*, *bla_TEM_* and *tet(A*) genes [33]. For the cluster 14 strains, all were positive for the IncB/O/K/Z and IncFII replicon types, and five (50%) were positive for all resistance genes identified in the outbreak strain from the UK.

We also identified a small cluster of high-virulent, MDR O80:H2 STEC (*n*=4). This strain has been reported as an emerging serotype in France and Western Europe, and studies have documented its association to bacteraemia and development of HUS [34–36]. Most of the O80:H2 strains have also been reported as MDR, commonly harbouring the resistance genes *bla*_TEM-1_, *sul*2 and *aph*-variants *StrA* and *StrB*, *drfA* and *tet(A*) [34–36]. This is in concurrence with our findings, where all O80:H2 strains were shown to carry *bla*_TEM-1B_, *sul*2, *aph*(3')*-Ia*, *StrA* and *StrB*, tet(A) and *drfA5* (in three of the four strains). Additionally, all strains were positive for the IncQ1, IncFII and IncB/O/K/Z replicon types. Additionally, one of the cases with an O80:H2 STEC infection was associated with travel to France. These observations indicate there might be an international spread of MDR STEC strains. As MDR STEC isolated in Norway share similar characteristics to those reported abroad, it is likely to assume that they may have a common epidemiological source and may respond to similar treatment alternatives. A study from France reported antibiotic treatment of some of the STEC O80:H2 cases *in vitro* with azithromycin with promising results [34].

STEC infections with high-virulent strains should be the primary targets for treatment. Based on successful clinical outcome and *in vitro* studies, azithromycin, ciprofloxacin, gentamicin, meropenem and fosfomycin have all been suggested or used for treatment of STEC infections [8, 9, 11, 12, 14–16, 37]. However, the use of antimicrobials against infections with STEC should not only take into consideration the potential for induction of Stx, but also selection and spread of AMR. As suggested, internationally dispersed strains harbouring large resistant plasmid may play an important role in the dissemination of resistance in STEC. The most common replicon type we identified was IncF, which showed a significant association to beta-lactam resistance. IncF plasmids have previously been associated with CTX-M group 1 enzymes in Enterobacteriales and often encountered in clinical settings associated with virulence [38]. However, we found that IncQ1 replicons were positively associated with both genotypic resistance and MDR. IncQ1 plasmids are broad-host range plasmids, which suggests that they are adapted to different hosts and have the potential to spread easily. Often they are reported to encode resistance against streptomycin (*strA*, *strB*) and sulphonamide (*sul2*) [39].

Still, the low prevalence of genotypic resistance in STEC is promising and advocate the use of selected antibiotics when necessary to avoid possible fatal outcomes. The overall resistance pattern reported was similar for both high- and low-virulent strains. Only 14.1 % of the high-virulent strains were genotypic resistant, of which most carried resistance determinants against aminoglycosides. The prevalence of azithromycin (macrolide) resistance among the high-virulent strains was low (2.4%) and azithromycin may represent an empiric treatment alternative. Ciprofloxacin could also be effective against most high-virulent STEC and has shown positive treatment outcomes in some clinical studies, however many *in vitro* studies have shown a high Stx induction potential of ciprofloxacin [8, 9, 14, 16]. Antimicrobial susceptibility testing of strains before initiating antimicrobial treatment is preferable, however for severe infections urgency dictates empirical treatment.

In this study we could not identify novel resistance genes or mutations that were absent from the database at the time of query. Although screening was limited by the use of only one open access resource for resistance gene determination, the ResFinder database includes the most prevalent resistant determinant. Coupled with several studies showing a high correlation between genotype and phenotype resistance, we believe our results give a reasonably good representation of the prevalence of genotypic resistance in STEC in Norway [18, 24]. The low numbers of strains for each independent variable tested for association to genotypic resistance, and the low number of resistant strains, reduces the power of the presented tentative significant associations. This is highlighted by the range of the 95 % confidence intervals for the calculated odds ratios, and associations should therefore be interpreted with caution.

## Conclusion

The overall prevalence of genotypic resistance in clinical STEC in Norway is low (16.1%). Genotypic resistance is more prevalent in non-O157 strains compared to O157 strains, and not associated to high-virulent STEC. Resistance to antimicrobials suggested for treatment, especially azithromycin, is low and may present an empiric treatment alternative for severe STEC infections.

## Supplementary Data

Supplementary material 1
