# Supplementary material for: Prevalence of genotypic antimicrobial resistance in clinical Shiga toxin-producing Escherichia coli in Norway, 2018 to 2020
Source: J Med Microbiol. 2021 Dec 6;70(12):001454. doi: 10.1099/jmm.0.001454 (PMC8744279; doi:10.1099/jmm.0.001454)
Supplement: Supplementary material 1 [file jmm-70-1454-s001.pdf]

Table S1. Distribution of replicon types among all *Shiga toxin-producing Escherichia coli* (STEC), genotypic antimicrobial resistant (AMR) STEC and genotypic multi-drug resistant (MDR) STEC.

Replicon type	Any STEC (n=459)	% of all STEC	AMR STEC (n=74)	%of AMR STEC	MDR STEC (n=39)	% of MDR STEC
IncFIB(AP001918)	345	75.2	49	66.2	31	79.5
Col(pHAD28)	191	41.6	38	51.4	23	59.0
IncB/O/K/Z	174	37.9	54	73.0	30	76.9
IncFII,	157	34.2	29	39.2	23	59.0
IncFII(pCoo)	130	28.3	22	29.7	10	25.6
Col156	117	25.5	27	36.5	17	43.6
Col(MG828)	108	23.5	20	27.0	10	25.6
Col440I	93	20.3	22	29.7	16	41.0
Col(MP18)	57	12.4	15	20.3	1	2.6
IncFII(29)	37	8.1	12	16.2	1	2.6
IncFIA	30	6.5	4	5.4	1	2.6
IncFII(pHN7A8)	28	6.1	7	9.5	3	7.7
IncI1-I(Gamma)	28	6.1	7	9.5	2	5.1
IncFII(pRSB107)	24	5.2	15	20.3	12	30.8
IncFII(pSFO	24	5.2	0	0.0	0	0.0
IncFII(pSE11)	22	4.8	6	8.1	3	7.7
FIA(pBK30683)	19	4.1	2	2.7	2	5.1
Col8282	17	3.7	6	8.1	4	10.3
IncI2(Delta)	16	3.5	4	5.4	2	5.1
IncQ1	16	3.5	16	21.6	16	41.0
Col(BS512)	12	2.6	9	12.2	8	20.5
None	10	2.2	1	1.4	0	0.0
p0111	9	2.0	2	2.7	2	5.1
IncY	8	1.7	0	0.0	0	0.0
ColpVC	7	1.5	3	4.1	3	7.7
IncI2,	7	1.5	1	1.4	1	2.6
IncX1	6	1.3	3	4.1	3	7.7
IncX4	6	1.3	2	2.7	1	2.6
IncP1	5	1.1	2	2.7	0	0.0
pKPC-CAV1321	4	0.9	4	5.4	4	10.3
IncHI22	4	0.9	4	5.4	4	10.3
IncHI2A	4	0.9	4	5.4	4	10.3
IncFIB(H89-PhagePlasmid)	4	0.9	3	4.1	3	7.7
IncFIC(FII)	4	0.9	2	2.7	2	5.1
IncI(Gamma)	3	0.7	0	0.0	0	0.0
Col(MGD2)	3	0.7	1	1.4	0	0.0
IncFIB(S)	3	0.7	2	2.7	2	5.1
IncFIB(pB171)	3	0.7	2	2.7	0	0.0
Col(VCM04)	2	0.4	0	0.0	0	0.0
Col440II	2	0.4	2	2.7	2	5.1
IncFII(S)	1	0.2	1	1.4	1	2.6
pXuzhou21	1	0.2	0	0.0	0	0.0

Table S2. Univariable statistical analysis of significant associations of replicon type, serotype and virulence genes to different genotypic resistance outcomes in Shiga toxin-producing *Escherichia coli* (STEC) Norway 2018-2020.

	Variable	Resistance outcome	Cases [a] N	Cases [a] Attack rate	Controls [a] N	Controls [a] Attack rate	Odds Ratio	95%CI
Inc-group	IncQ1	Aminoglycoside	16	33.3 %	0	0.0 %	-	-
	IncQ1	Beta-lactam	16	53.3 %	0	0.0 %	-	-
	IncQ1	Sulphonamide	16	40.0 %	0	0.0 %	-	-
	IncQ1	Tetracycline	15	42.9 %	1	0.2 %	317.3	[42.96-13377.68]
	IncQ1	Macrolide	5	45.5 %	11	2.5 %	33.1	[6.70-149.54]
	ColBS512	Macrolide	4	36.4 %	8	1.8 %	31.4	[5.45-152.91]
	IncFIIpRSB10	Macrolide	6	54.6 %	18	4.0 %	28.7	[6.46-127.79]
	ColBS512	Sulphonamide	8	20.0 %	4	1.0 %	25.9	[6.42-121.95]
	IncQ1	Quinolone	10	27.0 %	6	1.4 %	25.7	[7.65-91.12]
	IncFIIpRSB10	Sulphonamide	12	30.0 %	12	2.9 %	14.5	[5.37-38.60]
	IncFIIpRSB10	Tetracycline	11	31.4 %	13	3.1 %	14.5	[5.22-38.90]
	ColBS512	Beta-lactam	5	16.7 %	7	1.6 %	12.1	[2.77-47.20]
	IncFIIpRSB10	Quinolone	10	27.0 %	14	3.3 %	10.8	[3.86-28.74]
	IncFIIpRSB10	Beta-lactam	8	26.7 %	16	3.7 %	9.4	[3.10-26.17]
	IncBOKZ	Sulphonamide	32	80.0 %	142	33.9 %	7.8	[3.39-20.03]
	IncBOKZ	Beta-lactam	23	76.7 %	151	35.2 %	6.1	[2.43-17.02]
	IncFII	Beta-lactam	19	63.3 %	138	32.2 %	3.6	[1.59-8.69]
Serotype	O146:H28	Quinolone	11	29.7 %	0	0.0 %	-	-
	O111:H8	Macrolide	3	27.3 %	3	0.7 %	55.6	[6.16-459.67]
	O111:H8	Tetracycline	4	11.4 %	2	0.5 %	27.2	[3.67-305.87]
	O111:H8	Quinolone	3	8.1 %	3	0.7 %	12.3	[1.57-94.36]
	O111:H8	Sulphonamide	3	7.5 %	3	0.7 %	11.2	[1.44-85.94]
	O111:H8	Aminoglycoside	3	6.3 %	3	0.7 %	9.1	[1.77-69.06]
Virulence gene	capU	Sulphonamide	12	30.0 %	3	0.5 %	89.4	[18.08-836.38]
	sigA	Aminoglycoside	12	25.0 %	2	0.5 %	68.2	[14.06-636.04]
	sigA	Sulphonamide	12	30.0 %	2	0.7 %	59.4	[14.54-338.08]
	sigA	Tetracycline	10	28.6 %	4	0.9 %	42.0	[10.90-191.80]
	capU	Tetracycline	10	28.6 %	5	1.2 %	33.5	[9.38-131.93]
	sigA	Macrolide	4	36.4 %	10	2.2 %	25.0	[4.51-116.35]
	capU	Macrolide	4	36.4 %	11	2.5 %	22.7	[4.15-103.35]
	capU	Beta-lactam	8	26.7 %	7	1.6 %	21.9	[6.20-76.79]
	iroN	Beta-lactam	6	20.0 %	7	1.6 %	15.1	[3.81-56.22]
	iroN	Tetracycline	6	17.1 %	7	1.7 %	12.3	[3.16-45.45]
	capU	Quinolone	7	18.9 %	8	1.9 %	12.1	[3.43-40.57]
	iroN	Sulphonamide	6	15.0 %	7	1.7 %	10.4	[2.69-37.99]
	sigA	Quinolone	6	16.2 %	8	1.9 %	10.0	[2.66-34.96]
	sigA	Beta-lactam	5	16.7 %	9	9.3 %	9.3	[2.26-33-57]
	capU	Aminoglycoside	11	22.9 %	4	1.0 %	8.2	[8.31-134.40]
	iroN	Quinolone	5	13.5 %	8	1.9 %	8.1	[1.95-29.70]
	espP	Phenicol	8	72.7 %	126	28.1 %	6.8	[1.60-40.32]

[a]) For all association calculations between the independent variables and exposure characteristics: A case was defined as a STEC isolate positive for the independent variable and positive for the assessed characteristic. A control was defined as a STEC isolate positive for the independent variable and negative for the assessed exposed characteristic.

Table S3. Multivariable statistical analysis of significant associations of replicon type, serotype and virulence genes to single and multi-drug resistant outcomes in Shiga toxin-producing *Escherichia coli* (STEC) Norway 2018-2020.

Variable	Outcome[a]	Adjusted odds ratio (aOR)	95%CI
IncQ1	AMR	-[b]	-[b]
O146:H28	AMR	-[b]	-[b]
sigA	AMR	15.5	[1.61-147.94]
capU	AMR	9.4	[1.46-60.20]
IncFIIpRSB10	AMR	6.9	[2.19-21.53]
iroN	AMR	5.2	[1.45-18.70]
IncBOKZ	AMR	3.9	[2.10-7.16]
IncFII29	AMR	3.3	[1.49-7.47]
IncQ1	MDR	-[b]	-[b]
capU	MDR	19.8	[3.02-129.63]
sigA	MDR	12.3	[1.77-85.88]
iroN	MDR	11.3	[3.01-42.52]
IncFIIpRSB10	MDR	5.4	[1.40-21.10]
IncBOKZ	MDR	3.6	[1.54-8.34]

[a]Resistance to any class of antibiotics. AMR: genotypic antimicrobial resistance, MDR: multi-drug resistance.
[b] For columns with no outcome all STEC harbouring this variable were AMR/MDR.

Table S4. Distribution of serotypes, sequence types and number of single and multi-drug resistant strains within phylogenetic clusters of Shiga toxin-producing *Escherichia coli* (STEC) (n=318) in Norway 2018-2020.

Clusterd[d]	Total	Serotypes	Sequence Types (STs)	No. High-virulent	No. Resistant	No. Multi-drug resistant
1[a]	64	O157:H7	ST-11	64	5	5
2[a]	45	O26:H11	ST-21 (n=34),<br>ST-29 (n=10)	22	8	4
3[b]	39	O103:H2 (n=31),<br>O153:H2 (n=16),<br>O75:H2 (n=1),<br>O71:H2 (n=1),<br>O?:H2 (n=1)	ST-17 (n=37),<br>ST unknown (n=2)	5	2	1
4[b]	27	O128ac:H2 (n=12),<br>O128ab:H2 (n=14),<br>O?:H2 (n=1)	ST-4748 (n=3),<br>ST-25 (n=22),<br>ST unknown (n=2)	0	2	1
5[b]	24	O146:H21 (n=22),<br>O91:H21 (n=2)		1	1	1
6[b]	20	O142:H8 (n=5),<br>O?:H8 (n=15)	ST-26 (n=5),<br>ST-28 (n=14),<br>ST unknown (n=1)	0	0	0
7[b]	16	O63:H6 (n=16)	ST-583 (n=16)	0	0	0
8[a]	13	O145:H28 (n=11),<br>O?:H28(n=2)	ST-32 (n=11),<br>ST-137 (n=2)	11	1	0
9[b]	13	O91:H14 (n=12),<br>O?:H14 (n=1)	ST-33 (n=13)	0	2	1
10[b]	12	O113:H4 (n=9),<br>O38:H26 (n=1),<br>O118/O151:H12 (n=1),<br>O128ac:H10 (n=1)	ST-10 (n=11),<br>ST unknown (n=1)	3	2	1
11[b]	12	O146:H28 (n=11),<br>O?:H28 (n=1)	ST-738 (n=12)	1	12	0
12[b]	12	O20:H21 (n=2),<br>O?:H21 (n=6),<br>O88:H28 (n=2),<br>O187:H28 (n=2)	ST-40 (n=8),<br>ST-200 (n=4)	2	1	1
13[b]	11	O76:H19 (n=11)	ST-675 (n=11)	0	0	0
14[b, c]	10	O117:H7 (n=9), O?:H7 (n=1)	ST-504 (n=5),<br>ST-5292 (n=4),<br>ST-6880 (n=1)	0	10	10

[a] Statistically associated with high-virulent STEC

[b] Statistically associated with low-virulent STEC

[c] Statistically associated with genotypic antimicrobial resistance

[d] Clusters were defined as ≤500 allelic differences by core genome MLST and minimum 10 isolates per cluster.
